# Smoking Behaviour and Mental Health Disorders—Mutual Influences and Implications for Therapy

**DOI:** 10.3390/ijerph10104790

**Published:** 2013-10-10

**Authors:** Amedeo Minichino, Francesco Saverio Bersani, Wanda Katharina Calò, Francesco Spagnoli, Marta Francesconi, Roberto Vicinanza, Roberto Delle Chiaie, Massimo Biondi

**Affiliations:** 1 Department of Neurology and Psychiatry, Sapienza University of Rome, Rome 00185, Italy; E-Mails: bersani.fs@gmail.com (F.S.B.); wanda.katharina.cl@gmail.com (W.K.C.); francesco.spagnoli84@gmail.com (F.S.); francesconi.marta@hotmail.it (M.F.); r.dellechiaie@centrokahlbaum.it (R.D.C.); massimo.biondi@uniroma1.it (M.B.); 2 Department of Cardiovascular, Respiratory, Nephrologic and Geriatric Sciences, Sapienza University, Rome 00185, Italy; E-Mail: dott.vicinanza@gmail.com

**Keywords:** smoke, nicotine dependence, schizophrenia, mood disorders, anxiety, ADHD

## Abstract

Tobacco use is strongly associated with a variety of psychiatric disorders. Smokers are more likely than non-smokers to meet current criteria for mental health conditions, such as mood disorders, anxiety disorders and psychosis. Evidence also suggest that smokers with psychiatric disorders may have more difficulty quitting, offering at least a partial explanation for why smoking rates are higher in this population. The mechanisms linking mental health conditions and cigarette smoking are complex and likely differ across each of the various disorders. The most commonly held view is that patients with mental health conditions smoke in an effort to regulate the symptoms associated with their disorder. However some recent evidence suggests that quitting smoking may actually improve mental health symptoms. This is particularly true if the tobacco cessation intervention is integrated into the context of ongoing mental health treatment. In this paper we reviewed and summarized the most relevant knowledge about the relationship between tobacco use and dependence and psychiatric disorders. We also reviewed the most effective smoking cessation strategies available for patients with psychiatric comorbidity and the impact of smoking behavior on psychiatric medication.

## 1. Introduction

Cigarette smoking is associated with a wide range of adverse health effects, including several types of cancer, cardiovascular disease, dental disease, respiratory illness, reproductive problems, erectile dysfunction, disease of the eye, peptic ulcer disease and diminished bone health [1].

In the USA, the official leading causes of death are cardiac disease, cancer and stroke [2]. However, the actual causes of death are modifiable behavioral risk factors [3]; among these, tobacco use is the leading cause of death (18%), followed by poor diet and lack of physical activity (15%) and alcohol consumption (4%).

Smokers are more likely than non-smokers to meet current diagnostic criteria for mental health conditions, such as mood disorders, anxiety disorders and psychosis [4,5], and individuals with psychiatric disorders are far more likely than general population to smoke cigarettes. Adults in the United States with depression are about twice as likely to smoke in comparison to healthy people (43% *vs*. 22%) [6]. It has been estimated that those with one or more current psychiatric conditions smoke nearly half (44%) of all cigarettes consumed in the United States. Evidence also suggest that smokers with psychiatric disorders may have more difficulty quitting, offering at least a partial explanation for why smoking rates are higher in this population [7].

The mechanisms linking mental health conditions and cigarette smoking are complex and likely differ across each of the various disorders. The most commonly held view is that patients with mental health conditions smoke in an effort to regulate the symptoms associated with their disorder. However some recent evidence suggests that quitting smoking may actually improve mental health symptoms [8,9]. This is particularly true if the tobacco cessation intervention is integrated into the context of ongoing mental health treatment. However, more serious psychiatric disorders may require a more intensive intervention with frequent and long treatment sessions [10].

The aim of the present paper is to review and summarize the most relevant knowledge about the complex relationship between tobacco use and dependence and the main psychiatric disorders.

## 2. Methods

Relevant literature was identified through a search on Medline and PubMed. Search terms included: tobacco, nicotine abuse, nicotine addiction, schizophrenia, ADHD, mood disorders, anxiety, smoking behaviour, psychopharmacology, nicotinic acetylcholine receptors.

The majority of the studies about nicotine dependence are related to cigarette consumption.In medical scientific literature there is a lack of information about the newly emerging alternative tobacco products such as electronic cigarette, hookahs, beedis and kreteks. For this reason this review focuses only on cigarettes consumption.

References of selected citations were searched manually retrieve articles not found in the electronic database search. We included in our study several clinical trials, reviews and meta-analysis. A systematic review was not conducted during the proofreading period.

## 3. Nicotine Dependence

Nicotine dependence is characterized by tolerance and withdrawal symptoms that are associated with the pharmacological effects of nicotine.

The majority of the studies about nicotine dependence are related to the consumption of tobacco products. Although tobacco products contain over 4,000 chemicals [11], some of which could contribute to dependence, nicotine is the major dependence-forming constituent [12]. When using tobacco products, nicotine rapidly distributes from the lungs to the brain [13,14] where it interacts with high-affinity nicotinic acetylcholine receptors (nAChRs), resulting in rapid, pulsatile increases in the release of several neurotransmitters, notably dopamine in the mesolimbic system [15,16]. This neuronal pathway is thought to be important in the development of dependence to nicotine and other drugs of abuse, as the dopamine-induced activation engenders feelings of reward and positive reinforcement [17,18]. Reward and positive reinforcement initiate nicotine-seeking behaviors that can become sustained upon repeated nicotine exposure since long-term systemic exposure to nicotine leads to pharmacologic tolerance to some of its effects involving up-regulation of nAChRs.

In addition to the mesolimbic dopamine pathway, other neurotransmitter systems are likely involved in the reinforcing effects of nicotine, such as glutamatergic and GABAergic neurons that contain nAChRs, which nicotine can activate or desensitize, and possibly up-regulate [19,20]; moreover, interactions between nAChRs and the opioid system have been reported to play a role [21,22].

The effects of nicotine are in part mediated via nAChRs, ligand gated ion channels that mediate fast synaptic neurotransmission [23]. nAChRs are grouped into muscle-type and neuronal receptors. Neuronal nAChRs are composed of α (α2–α10) and β (β2–β4) subunits. These subunits can assemble to form heteropentamers from combinations of α2, α3, α 4, α6 with either β2 or β4, or with the addition of α5 and/or β3 subunits. Neuronal nAChRs are predominantly found on presynaptic nerve terminals where they modulate the release of neurotransmitters.

The different subtypes of neuronal nAChRs display a complex expression profile in the brain [24]. Nicotinic receptors containing α4 and β2 subunits (denoted as α4β2 nAChRs) are the most prevalent in the central nervous system (CNS) [25] and are considered the major subtype involved in regulating the addiction-relevant actions of nicotine [26]. Indeed, nicotine-replacement therapy (NRT), such as nicotine gum and patch, is believed to act primarily at high-affinity α4β2 nAChRs [27], and medications such as varenicline were developed as α4β2 nAChR partial agonists. Hence, there is much effort devoted to developing nAChR-based therapeutics for smoking cessation. There is now considerable evidence that the reinforcing actions of nicotine are related to its stimulatory effects on mesoaccumbens dopamine transmission, which comprises dopamine-containing neurons that arise in the ventral tegmental area (VTA) and project to the nucleus accumbens (NAcc). In particular, the actions of nicotine at α4β2 nAChRs in the VTA are believed to play a key role in the reinforcing effects of the drug that motivate the establishment and maintenance of the tobacco habit.

The clinical utility of nicotine NRT is believed to reflect an action at high-affinity α4β2 nAChRs by the nicotine in these products, thereby substituting for at least some of the actions derived from nicotine in tobacco smoke. These findings suggest that modulation of midbrain dopamine systems may, to some degree at least, represents a common underlying mechanism of currently available smoking-cessation agents.

Although α4β2 nAChRs are undoubtedly involved in nicotine reinforcement, there is growing evidence for contributions from other subtypes of nAChRs. In particular, α6 nAChRs are emerging as an important class of nAChRs in nicotine reinforcement. There is particularly dense expression of α6 subunit mRNA in the VTA and NAcc [28], which plays a major role in supporting the positive reinforcing actions of nicotine. Moreover, α7 nAChRs seem to be involved in regulating the stimulatory effects of nicotine on midbrain dopamine systems and, with α6, may represent a novel target for medications development [29].

When smoking, nicotine reinforces addictive behaviors by stimulating the mesolimbic reward pathway. During abstinence, withdrawal symptoms such as depressed mood, insomnia, and irritability [30] are hypothesized to occur because target nAChRs are either no longer occupied by nicotine or change from the desensitized to the active state and become responsive to nicotine, once more causing a strong urge to resume smoking in order to alleviate these negative feelings [31].

## 4. Schizophrenia

Schizophrenia is a chronic and severe mental illness affecting approximately one per cent of the general population [32].

A meta-analysis of 42 epidemiological studies across 20 different countries showed that people with schizophrenia have more than five times the odds of current smoking than the general population, and smoking cessation rates are much lower in smokers with schizophrenia compared with the general population [33]. In addition, smokers with schizophrenia smoke more heavily and extract more nicotine from each cigarette [33,34].

People with schizophrenia have a shorter life expectancy than the general population, and chronic cigarette smoking has been suggested as a major contributing factor to higher morbidity and mortality from malignancy and cardiovascular and respiratory diseases in this group of patients, especially in people aged 35 to 54 years [35,36].

There may be a shared neurobiology between the deficits observed in schizophrenia and drug dependence, because both implicate altered dopaminergic and cholinergic transmission in the mesolimbic systems [37,38].

Cigarette smoking transiently normalizes an abnormal auditory sensory (P50) gating mechanism in schizophrenic patients [39]. It is suspected that this relates clinically to the subject’s perception of having an auditory hallucination as well as the ability to filter out other distracting noises. This electrophysiological abnormality has been linked to decreased hippocampus size in schizophrenics and a reduction in α7 nicotinic receptors on GABA-B inhibitory interneurons [40]. Abnormalities in P50 inhibition are also found in asymptomatic family members who do not have schizophrenia, and infrequently in the general population [41]. Interestingly the P50 defect in these family members is reversed by a dose of nicotine, making the P50 system an interesting model to examine α7 nicotinic receptors in humans. Pedigrees of these families reveal an autosomal dominant pattern of inheritance for this abnormality, linked to chromosome 15q13-14, the site of the α7 nicotinic receptor [42,43,44].

## 5. Attention Deficit Hyperactivity Disorder (ADHD)

Research using clinical samples indicates that individuals with ADHD smoke at rates that are significantly higher than those of the general population and/or non-diagnosed controls among both adults (41%–42% *vs.* 26% for ADHD and non-ADHD, respectively) and adolescents (19.0%–46% *vs.* 10%–24% for ADHD and non-ADHD, respectively) [45,46,47,48]. In a population-based sample of over 15,000 young adults, a linear relationship was identified between the number of retrospectively self-reported ADHD symptoms and the lifetime risk of regular smoking [49] .This study also found a negative association between the number of ADHD symptoms and the age of onset of smoking. Among current smokers, it identified a positive association between the number of ADHD symptoms and number of cigarettes smoked per day.

Both ADHD and smoking are highly heritable; genetic factors account for 60%–80% and 56% of the two phenotypes respectively [50,51]. Candidate gene studies have identified a number of similar genetic markers associated with both ADHD and smoking phenotypes, suggesting that several common neurobiological mechanisms may give rise to this comorbidity [52,53,54,55].

From a neuropharmacological perspective, ADHD is hypothesized to be the result of an aberrant striatal dopaminergic system that results in disrupted dopaminergic transmission in corticostriatal circuits. These disruptions, in turn, give rise to the characteristic deficits in executive functioning observed in ADHD patients [56,57]. This altered dopaminergic hypothesis is supported by studies showing differences in dopamine transporter (DAT) density in relevant striatal areas in ADHD patients compared with controls [58,59,60]. Although these studies have reported discrepant findings with respect to the direction of DAT density change (*i.e.*, some report higher levels, and some report lower levels in ADHD), collectively they suggest associations of DAT density and its consequent effects on DA neurotransmission with the clinical condition of ADHD [61].

## 6. Anxiety Disorders

The prevalence of nicotine dependence is higher among individuals with any anxiety disorder than in the general population, with data indicating that the percentage of current smokers varies among anxiety disorders, ranging from 31.5% for social phobia to 54.6% for generalized anxiety disorder [62].

Cross-sectional studies have uniformly indicated that smoking, compared with not smoking, is associated with more panic relevant symptoms and impairment among both clinical [63,64] and nonclinical samples [65]. Little research has directly examined mechanisms underlying this association, and the possible links are likely multifactorial. The association may be due to shared common predisposing factors, such as genetic predisposition or a tendency to experience negative affect states, placing individuals at risk for both anxiety disorders and nicotine dependence [66,67]. Generalized personality-based factors may be relevant to understanding the relationship of panic attacks and smoking; however, it is unclear if specific panic relevant individual differences (e.g., anxiety sensitivity) or social-environmental factors play similar roles.

There may be important neurobiological mediators of the comorbidity between PTSD and nicotine dependence [68]. The hypothalamic-pituitary-adrenal (HPA) axis is a system that is involved in the development of nicotine tolerance. Cortisol, a critical peripheral HPA hormone [69], is produced in response to stressful events [70] and has been shown to respond to nicotine administration and deprivation [71,72]. Alterations in the HPA axis have been associated with PTSD [68,73], and some of these alterations may increase the risk for smoking dependence. Rasmusson [68] has hypothesized that cortisol elevations induced by various factors (e.g., stress, conditioned contextual cues) enhance both tolerance and sensitization to various effects of nicotine, and may help account for increased smoking in those with PTSD and other anxiety disorders. Increased HPA axis activity may result from a genetic predisposition, stress, trauma exposure, or a gene-environment interaction. Investigations that include genetic, environmental, and neurobiological information may be useful in understanding the relationship between anxiety disorders and smoking. People with anxiety disorders may initiate and maintain smoking behavior in an attempt to self-regulate or cope with adverse emotional distress [74,75]. This model predicts that individuals with premorbid anxiety risk factors or full-blown disorders are more apt to smoke to regulate emotional states [74,76,77].

## 7. Bipolar Disorder

Estimates of the prevalence rate of current smoking among individuals with BD range from 30% to 70% [62,78,79,80]. Although some of this variability can be attributed to small sample sizes and non-representative clinical samples, even population-based prevalence studies have produced disparate findings. For example, data from the 1992–1993 National Comorbidity Survey (NCS) suggested that the prevalence of smoking in BD was 69% [62], whereas the more recent 2007 National Health Interview Survey (NHIS) showed a prevalence rate of 46% [79]. Despite this variability in prevalence estimates, controlled population-based [62,79,81] and clinical studies [82,83,84] conducted in the U.S. and Europe have consistently demonstrated that the prevalence of smoking is approximately two to three times higher among individuals with BD than in the general population.

Mechanisms underlying the relationship between smoking and BD are likely complex and multifactorial, including genetic and environmental factors as well as their interactions. Research on these mechanisms is sparse in BD, but several decades of research on the relationship between depression and smoking suggest causal pathways in both directions [85,86] and that these relationships may also be explained by common or correlated risk factors [87]. One potential explanation for the high rate of smoking among individuals with BD is that symptoms of BD may increase the risk of initiating or maintaining regular smoking. Because of tobacco smoke monoamine oxidase (MAO)-inhibiting effects [88] and nicotine capacity to stimulate the release of neurotransmitters that improve mood and induce feelings of pleasure (e.g., serotonin and dopamine), the self-medication hypothesis has been invoked to explain the greater risk of smoking among individuals with depression. Another possible explanation for the high rates of smoking in BD is that smoking may increase the risk of developing BD; for example, smoking may lead to alterations in neurophysiology that unmask an underlying vulnerability to affective episodes. Consistent with this hypothesis, there is evidence from both preclinical and clinical studies to suggest that chronic exposure to nicotine may increase risk of developing depression by desensitizing nicotinic acetylcholine receptors in the brain’s limbic system [89]. It has also been proposed that longer-term exposure to nicotine can induce depression through actions on serotonin pathways in the hippocampus, but smokers are protected from these effects as long as they continue to smoke [90].

Although there has been no systematic study of this phenomenon as it relates to onset of BD, it has been suggested that smoking cessation may precipitate an affective episode in some smokers who have already been diagnosed with BD [83].

BD and smoking may also be linked through common risk factors, including both genetic and environmental influences. Regarding shared genetic risks, several overlapping candidate genes for BD and smoking have been identified, including genes that encode: (i) catechol-*O*-methyltransferase (COMT); (ii) the dopamine transporter; and (iii) the serotonin transporter [91]. Moreover, a number of neurochemical systems have been implicated in both BD and nicotine dependence, with most attention focused on dopamine, serotonin and norepinephrine [92,93,94,95].

## 8. Depression

Cigarette use is disproportionately higher among people with depression than among people in the general U.S. population. Cross-sectional studies report that over 30% of patients with current depression are daily smokers [95,96]. Nearly 60% with a lifetime history of depression are current or past smokers [62]. Conversely, smokers, as compared with nonsmokers, have significantly higher rates of lifetime depression [97]. Smokers who are nicotine dependent are twice as likely as nonsmokers to have a history of depression [98]. The lifetime prevalence of major depression is high among smokers in clinic based smoking treatment, with rates as high as 64% [99].

Several studies suggest a genetic predisposition to both nicotine dependence and depression. Family studies reveal smoking patterns that differ according to the subtype of depressive disorder, with the closest association observed between dysthymia and heavy smoking [100].

Clinical observations of mood changes and depression linked to smoking led to the investigation of antidepressants to treat nicotine dependence. The only FDA-approved non-nicotine medication for nicotine dependence is bupropion, believed to be effective because of its dopaminergic activity on the pleasure and reward pathways in the mesolimbic system and NAcc and because it acts as a noncompetitive nicotinic receptor antagonist [31,101]. Nortriptyline, which also has dopaminergic and adrenergic properties, has been found to be effective, independent of depression history and can reduce the negative affect related to quitting [102]. In addition to the effects of nicotine, tobacco contains monoamine oxidase (MAO) inhibitors, which cross the blood–brain barrier and inhibit the enzyme needed to break down norepinephrine, serotonin, and dopamine in the synaptic cleft. The result is to enhance the actions of these neurotransmitters in a manner similar to antidepressant treatment [18].

## 9. Impact of Smoking Behavior on Psychiatric Medications

Smoking impacts the course of psychiatric disorders through its profound effect on the metabolism of psychotropic drugs and is thus a contributory factor to the individual variations observed in drug responses [103].

Nicotine metabolism is mediated primarily by the cytochrome P450 1A2 (CYP1A2) and by CYP2A6. Since many psychiatric drugs, including diazepam, haloperidol, olanzapine, clozapine, fluphenazine, and mirtazapine, are also metabolized through CYP1A2 induction, smoking can lower their therapeutic blood levels and decrease their effectiveness [104,105]. For example, a daily consumption of five cigarettes is sufficient for the induction of olanzapine metabolism and decreased plasma olanzapine concentrations, while heavy smokers would require a 50–100% increase in olanzapine dose compared with nonsmokers to achieve the same therapeutic level [103].

Smoking cessation leads to increased plasma concentrations with increased risks of adverse effects, creating a requirement for close drug dose monitoring in smokers during smoking cessation [103].

Conversely, antipsychotic medications may differentially impact an individual’s smoking status; for example, patients with schizophrenia were found to smoke more after initiation of haloperidol treatment and less when switched from haloperidol to clozapine [106,107]. Other atypical antipsychotic medications, for example olanzapine and risperidone, can also reduce smoking rates [106,108,109,110] and this effect may be attributable to increased cortical dopamine release [111,112], as well as enhanced prefrontal NMDA receptor-mediated transmission [113].

Therefore, nicotine use has several clinically relevant implications for patients taking antipsychotics, and close monitoring of these patients is highly recommended [10].

## 10. Treating Tobacco Use and Dependance

As psychiatric patients are often excluded from randomized controlled trials, there are not definitive data about the most effective treatment for nicotine dependence for smokers with psychiatric comorbid conditions. This has been due, in part, to concerns that smoking cessation may lead to an exacerbation of psychiatric symptomatology and an erroneous belief that smokers with comorbid psychiatric conditions are not motivated to quit smoking. Fortunately, the preponderance of evidence suggests that psychiatric symptoms typically do not worsen and, in fact, may even improve following abstinence from tobacco [114]. Therefore, clinicians should nor refrain from addressing nicotine dependence in patients with comorbid psychiatric disorders, but rather should encourage cessation and provide support as they would with any other smoker. Nevertheless, considering that a quit attempt could act as a stressor with the potential to temporarily affect symptomatology in the same way as any other stressful event [115], monitoring of patients’ psychiatric status during the quitting process is warranted.

To date, varenicline is the only FDA-approved smoking-cessation agent that was rationally designed through traditional drug discovery processes based on its action as an α4β2 nAChR partial agonist [116]. It is important to note, however, that varenicline is also a full agonist at α7 nAChRs [117]. The rationale for developing a α4β2 nAChR partial agonist for smoking cessation was twofold. First, it was hypothesized that a partial agonist may competitively bind to α4β2 nAChRs and thereby attenuate the stimulatory effects of nicotine obtained from tobacco smoke on mesoaccumbens dopamine transmission [118]. The stimulatory effect of nicotine on midbrain dopamine transmission is considered central to its reinforcing properties that contribute to the development and maintenance of the tobacco habit [119]. Hence, attenuation of this effect by varenicline may decrease the reinforcing effects of nicotine thereby aiding smoking cessation efforts. Second, it was hypothesized that the intrinsic low levels of activation of α4β2 nAChRs by a partial agonist may substitute for the stimulatory effects of nicotine on mesoaccumbens dopamine transmission during abstinence, eliciting a moderate and sustained increase in dopamine levels [118]. In this manner, α4β2 nAChR partial agonists may counteract decreases in mesoaccumbens dopamine transmission believed to occur during abstinence from tobacco smoking [27], thereby again facilitating smoking-cessation efforts [118]. Hence, FDA-approved drugs that modulate midbrain dopamine transmission, such as aripiprazole [120], may be particularly promising candidate for repurposing as smoking-cessation agents. Bupropion is the only FDA-approved smoking-cessation aid that does not target nAChRs as its primary mode of action. Instead, bupropion is an atypical antidepressant used for the treatment of depression. Nevertheless, it is important to note that bupropion and its metabolites can act as nAChR antagonists [121]. The ability of bupropion to facilitate smoking cessation was discovered serendipitously when it was shown to decrease cigarette consumption in depressed patients. The precise mechanism through which bupropion facilitates abstinence is unclear but nevertheless raises the intriguing possibility that other FDA-approved medications could likewise facilitate smoking cessation through novel mechanisms of action.

Tolcapone, an inhibitor of the COMT enzyme involved in degradation of catecholamine neurotransmitters, is FDA-approved for the treatment of the symptoms of Parkinson’s disease when used in conjunction with L-dopa. Tolcapone is also currently in clinical trials for smoking cessation [122], the outcome of which will be particularly intriguing considering that genetic variation in COMT is associated with increased risk of tobacco dependence [123]. Interestingly, an ongoing trial [122], is assessing the effects of the antihistamine meclizine, used to treat motion sickness, on smoking cessation. Recently, blockade of H1 histamine receptors was shown to decrease intravenous nicotine self-administration in rats, supporting the notion that FDA-approved drugs that modify histaminergic transmission may indeed serve as novel smoking-cessation agents [29].

The acetylcholinesterase (AChE) inhibitor galantamine, approved for the treatment of cognitive symptoms of Alzheimer’s disease, reduced the number of cigarettes smoked in alcohol-dependent patients [124]. This suggests that boosting endogenous cholinergic transmission may be an effective strategy to facilitate smoking cessation and abstinence [29].

A growing trend in clinical trials has been the testing of compounds that do not require FDA approval as they are classified as food supplements, such as S-adenosyl-L-methionine (SAMe), l-acetylcarnitine, *N*-acetyl-cysteine (NAC) and dehydroepiandrosterone (DHEA) [125]. SAMe is a methyl donor involved in the synthesis of monoamine neurotransmitters and has been reported to have utility for the treatment of depression [126]. It has been reasoned that increasing monoamine neurotransmitter levels through consumption of SAMe, particularly during withdrawal from tobacco smoking when dopamine transmission is hypothesized to decline, may attenuate the aversive effects of tobacco withdrawal and thereby facilitate long-term abstinence. However, no reports on the effects of SAMe on smoking abstinence have appeared in the published literature. In preclinical models of drug dependence, NAC normalizes otherwise decreased levels of basal glutamate in the accumbens and attenuates cue-evoked drug-seeking behaviors [127]. Moreover, NAC reduced the number of cigarettes consumed by smokers [128]. Hence, it is possible that NAC may be successfully used to facilitate smoking cessation. DHEA is a neuroactive steroid whose levels were shown to be inversely related to the magnitude of craving and negative affect in abstinent smokers and may predict nicotine-dependence severity [129]. Hence, boosting DHEA levels may decrease craving and negative affect during abstinence and thereby promote continued cessation of the tobacco habit. Based on these observations, it is possible that other compounds currently listed as food supplements may have beneficial effects on smoking cessation [29].

The antihypertensive medication clonidine, an α2 adrenergic agonist, is sometimes used as second-line agents for smoking cessation [130], but its use in this context has not been approved by the FDA.

A novel non-drug-based strategy to facilitate smoking cessation is nicotine vaccines. Nicotine-based vaccines can prime the immune system to recognize nicotine as foreign and to mount an immune response against the drug. In doing so, vaccines may reduce the amounts of nicotine penetrating into the brain [131]. Conceptually, a potential drawback related to the use of vaccines to treat tobacco dependence is the fact that smokers will often compensate for decreases in the actions of nicotine, as would be expected when a vaccine decreases concentrations of nicotine penetrating into brain tissues, by increasing their tobacco consumption to overcome this effect [132]. Other potential issues related to the successful use of vaccines include difficulties achieving sufficiently high antibody titers, the fact that vaccines are generally short lived, and significant interindividual variation in response to the vaccine typically observed [133].

Although current smoking-cessation agents facilitate cessation efforts, they have limited effectiveness. In smokers attempting to quit, about 23% treated with varenicline and 16% treated with bupropion remain abstinent after 1 year, compared with just 9% of those treated with placebo [134]. Pharmacotherapy is therefore an effective strategy to promote abstinence from smoking, but there remains considerable risk of release even when treated with the most efficacious medications currently available.

## 11. Treating Tobacco Use and Dependance in Patients with Psychiatric Comorbidities

### 11.1. Schizophrenia

Patients with schizophrenia are the group with the highest rates of tobacco use (70-85%), and for whom there has been considerable interest in identifying effective smoking cessations interventions [18]. Between the recommended pharmacotherapies for treating nicotine dependence, bupropion has been demonstrated to have the greatest benefits for these patient population [135,136].

A recent Cochrane review of smoking cessation interventions in individuals with schizophrenia found seven trials that compared bupropion with placebo and a meta-analysis of these trials showed that smoking cessation rates were significantly higher after bupropion *vs.* placebo at the end of treatment and after 6 months [44]. Bupropion did not lead to significant differences in positive, negative, or depressive symptoms compared with placebo [44,136]. The same meta-analysis found there was no evidence of benefit of NRT in smokers with schizophrenia, although there were very few published trials available for evaluation [44]. However, abstinence rates can be increased when bupropion or NRT are used in combination with atypical antipsychotics, such as either clozapine, compared with standard antipsychotics [137]. Many of the pharmacotherapy trials included in the Cochrane review also provided behavioral support (e.g., group cognitive behavioral therapy) to the participants but the studies did not directly compare different combinations of pharmacotherapy and behavioral therapy [31,44]. Although there is a post-marketing case report detailing exacerbated psychotic symptoms in a patient with schizophrenia being treated with varenicline for tobacco dependence [138], emerging clinical studies do not suggest that varenicline treatment worsens or exacerbates psychiatric symptoms. A small case series involving 19 smokers with schizophrenia, who had previously attempted cessation with NRT or bupropion, reported all patients experienced reduced cravings after receiving varenicline and none reported psychotic relapse, worsening of psychiatric symptoms, or side-effects of antipsychotic treatments [139]. Furthermore, in a small, open-label study involving 14 smokers with schizophrenia, varenicline demonstrated a beneficial effect on the cognitive dimensions of nicotine withdrawal [140], similar to effects observed in healthy smokers [141].

Finally, once abstinence is achieved in patients with schizophrenia, there will be a need to review the choice and dose of antipsychotics used, due to the effect nicotine has on drug metabolism, which may differentially impact antipsychotic medications [105]. Overall, approaches found to be effective in the general population are likely to be successful among patients with schizophrenia as well, a though it may be helpful to repeat treatment recommendations several time, make them more concrete, and extend the duration of treatment support [142]. As with any group of smokers, interventions that combine behavioral counseling and pharmacotherapy have the greatest probability of success.

### 11.2. Mood and Anxiety Disorders

Given that about 45% of patients with major depressive disorder smoke [7], considerable attention has also been focused on this population. In general, great levels of treatment support, appear to be particularly beneficial for smokers with a history of major depressive disorder [143]. At present, the approach with the most empirical support, is the inclusion of cognitive behavioral therapy focusing on mood management as part of the cessation intervention. Although the additional benefits for those of history of a single major depressive episode have been limited, cognitive behavioral therapy have been shown to significantly enhance outcomes among those with a history of recurrent episodes [144,145]. With regard to pharmacotherapy, there is insufficient evidence to suggest that a particular type of medication is more effective for patients with comorbid depression.

Despite the high rates of smoking among those with anxiety disorders, surprisingly little is known the best way to approach treatment for this patient population. Results for one small-scale study [146] suggest that bupropion may increase cessation rates among patients with PTSD.

There is not sufficient evidence to make definitive recommendations regarding how best to promote smoking cessation among patients with other anxiety disorders (e.g., generalized anxiety disorders, social phobia, panic disorder).

### 11.3. ADHD

Bupropion has shown efficacy in treating adults with ADHD and has also been approved by the Food and Drug Administration as an aid to smoking cessation [147,148,149]. Novel cholinergic agents have also shown promise in treating adults with ADHD [150,151]. Whether these agents would work for treating comorbid ADHD-smoking is largely unknown, although one open-label pilot study with adolescents reported positive results [152]. 

Non pharmacological approaches to treating ADHD might also be useful in facilitating smoking cessation. Emerging work shows promise for the use of cognitive-behavioral treatment of adults with ADHD. It would be important to evaluate whether these treatment approaches would serve as useful adjuncts to smoking cessation in those with ADHD [153]. 

## 12. Conclusions

It is reasonable to conclude that approaches found to be successful among the general population and that include a combination of behavioral counseling and pharmacotherapy are also likely to be effective with these groups, although abstinence rates may be slightly lower [154]. 

Overall the implication for clinicians is that they should not be hesitant to encourage mental health patients to quit smoking. Not only will it help improve their physical help, but it may also improve rather that exacerbate their mental health.
